# Ultrasound-Assisted Lumbar Puncture: A Narrative Review With Implications for Educators in the Clinical Neurosciences

**DOI:** 10.1212/NE9.0000000000200354

**Published:** 2026-10-01

**Authors:** Allison Grubman, Astra Yu, Nishitha Bujala, Carolin Dohle, Stephanie Gandelman, Adam M. Karp

**Affiliations:** 1 School of Medicine, New York Medical College, Valhalla, NY; and; 2 Department of Neurology, Westchester Medical Center, Valhalla, NY.

## Abstract

In this narrative review, we examine the evidence on ultrasound-assisted lumbar puncture (USLP) and evaluate its applicability for broader adoption in neurology. Lumbar puncture (LP) is essential for diagnosing many neurologic conditions. However, landmark-guided LPs can be technically challenging, with failure rates up to 28%, especially in patients with obesity, prior surgery, or anatomic variations. These difficulties can delay diagnosis and treatment of time-sensitive conditions. Ultrasound-assisted LPs (USLPs) have emerged as a possible method to improve success rates and reduce complications. Limited neurologic literature on USLP suggests this technique may remain understudied and underutilized in neurology, with lack of exposure and formal training contributing to slow adoption. Across multiple studies and meta-analyses, USLP increased first-attempt success rate, reduced traumatic LPs, shortened procedure time, and lowered pain scores. These benefits were particularly pronounced in patients with obesity or spinal anatomical variations. Studies that reported no significant difference between USLP and landmark-based LP tended to have limited sample sizes and variability in study design, including differences in operator experience and definitions of procedural success. Since referring LPs to radiology for fluoroscopic guidance is both costly and time consuming, incorporating USLP into neurology practice may allow neurologists to independently perform difficult LPs. Broader integration of USLP into neurologic practice can enhance bedside procedure success, decrease dependence on radiology, and strengthen neurologists' capacity for efficient, high-quality diagnostic procedures. Further studies should explore current practices surrounding LP training and the effect of USLP on clinical and system-level outcomes in neurology settings.

## Introduction

Lumbar punctures (LPs) are an essential diagnostic tool in neurology, particularly in the evaluation of suspected CNS infections, subarachnoid hemorrhage, and demyelinating disease.^1^ Despite their diagnostic value, LPs can be technically difficult in patients with obesity, prior spinal surgery, or other spinal anatomical variations. These anatomical challenges, often characterized as high body mass index (BMI), having spinal curvature, and degenerative changes, have been associated with failure rates ranging from 7.7% to up to 28% in outpatient neurology settings when LPs are performed using traditional landmark-based techniques.^2-4^ Failure reports have notably been reported as independent of provider training level.^3^ This is particularly concerning in neurologic emergencies where delayed LP can worsen outcomes.^5^ Furthermore, prolonged or repeated attempts can increase patient discomfort.

In recent years, ultrasound-assisted LPs (USLPs) have emerged as a possible method to improve first-attempt success rates, decrease periprocedural needle redirections, shorten procedure time, reduce pain scores, and lower the risk of traumatic LPs.^6-9^ The most common approach involves preprocedural ultrasound to identify and mark an optimal insertion site, although real-time ultrasound guidance can also be used.^10^ The preprocedural approach is generally preferred because it enables the use of both hands for needle insertion and is less resource-intensive, requiring fewer sterile materials. It is typically performed as follows: a curvilinear transducer is placed transversely across the lumbar spine to identify and mark the midline. The transducer is then rotated into the sagittal plane and placed in the midline or the paramedian location. From here, the sacrum and intervertebral spaces can be identified, the desired needle insertion site is marked, and depth from the skin to the ligamentum flavum is measured. All references to USLP in this review will refer to the preprocedural approach (Figure). A detailed description of the technique, including instructional videos, is provided by Millington et al.^11^ (2018).

**Figure:**
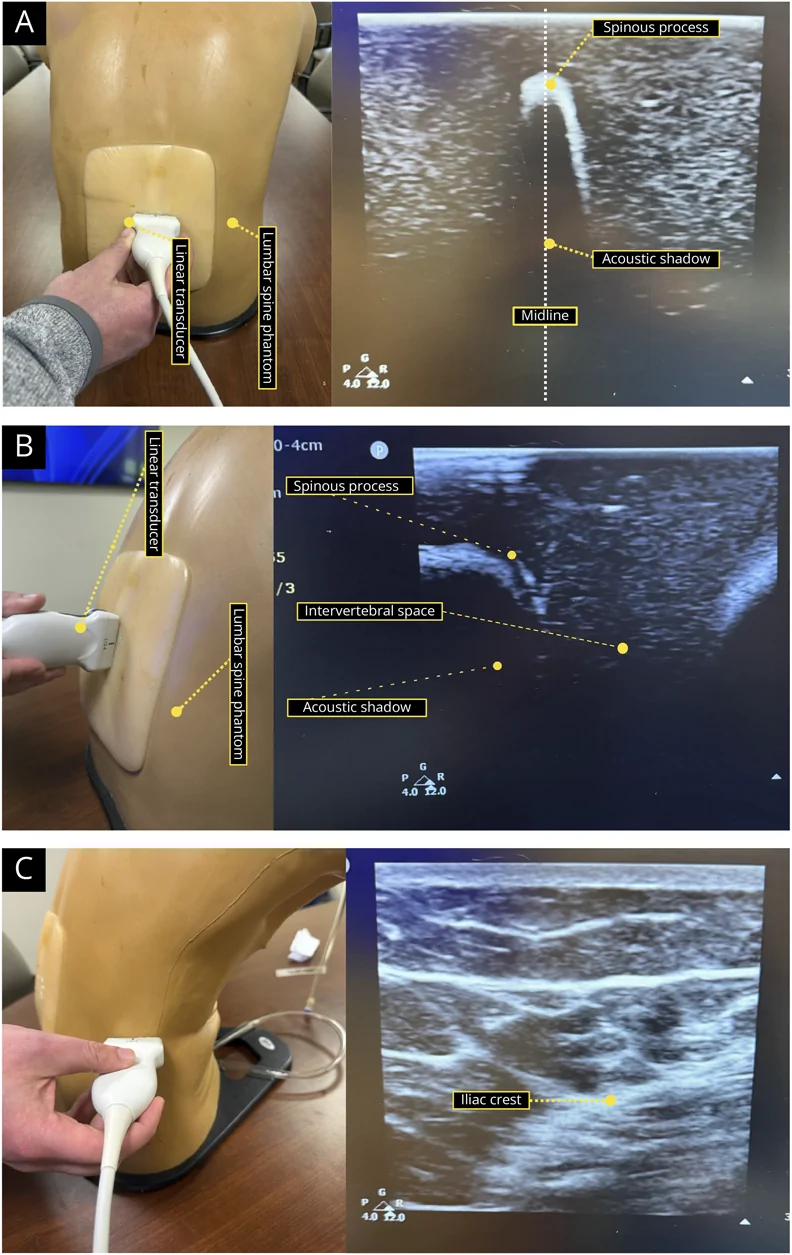
Representative Probe Positioning and Corresponding Ultrasound Images for USLP Simulation A US-compatible lumbar spine phantom can be used for simulation-based education of USLP. (A) Transverse probe positioning and US view on a US-compatible lumbar spine phantom for visualizing the midline. (B) Midsagittal probe positioning and US view on a US-compatible lumbar spine phantom for visualizing the intervertebral spaces. (C) Lateral probe positioning on a US-compatible lumbar spine phantom and corresponding US view on a human volunteer for visualizing the iliac crest. USLP = ultrasound-assisted lumbar puncture.

Randomized controlled trials (RCTs) in USLP are often conducted in emergency medicine^7,8^ and anesthesiology^12,13^ settings, with comparatively few randomized studies in neurology departments.^14^ Existing neurology-focused literature similarly highlights a lack of data in this setting,^14^ despite reported challenges in LP performance and training among neurology residents.^3,15^ This narrative review synthesizes the clinical evidence surrounding USLP in adult populations and explores the potential role of USLP in neurology training.

## Benefits of USLP

A growing body of evidence supports the clinical advantages of USLP. The first meta-analysis on USLP in 2013 which included randomized and quasirandomized trials reported a significantly lower risk of failed LPs using ultrasound assistance (RR = 0.19, 95% CI 0.07–0.56), with an absolute risk reduction of 12% and a number needed to treat (NNT) of 9. The ultrasound group also required fewer insertion attempts (MD = −1.01, 95% CI −1.56 to −0.45).^5^ These findings were supported by a meta-analysis of RCTs in 2019 which found that ultrasound-assistance was associated with an 8.9% increase in success rates, a 16.4% reduction in traumatic LPs, and a modest, yet statistically significant, reduction in time to successful puncture (MD = −1.80 minutes; 95% CI –3.57 to −0.03). Although this was statistically significant in the manuscript, this minor reduction in time is of uncertain clinical significance. They also reported a significant reduction of −2.53/10 in pain scores (95% CI –3.89 to −1.17).^7^ In addition, a recent meta-analysis of RCTs in 2024 found that using preprocedural ultrasound for neuraxial punctures increased first-pass success (RR 1.6, 95% CI 1.3–1.9), and increased patient satisfaction (SMD 0.28, 95% CI 0.092–0.47); however, the broad nature of procedures included in this meta-analysis may decrease the applicability of these data to USLP^16^

Recent retrospective studies add further support, showing that ultrasound assistance was associated with reduced odds of traumatic LPs (OR: 0.53, 95% CI 0.31–0.89, *p* = 0.02).^17^ Another group found that providing standardized training on a paramedian preprocedural ultrasound approach resulted in a 13.6 percentage-point increase in the probability of successful LP (95% CI 2.8–24.4, *p* = 0.014) with an odds ratio of 2.4 (95% CI 1.2–4.8).^18^ Collectively, these findings highlight the clinical value of USLP and its potential to improve procedural success and safety.

## Advantages of USLP in the Setting of Anatomic Variations

The clinical advantages of USLP are particularly pronounced in patients with anatomical variations, in whom landmark-based techniques are especially prone to failure. Procedural difficulty is multifactorial and reflects the combined influence of patient body habitus, spinal anatomical variation, and soft tissue characteristics. Increased central adiposity, in particular, is a well-established predictor of traditional LP failure,^3,17^ likely because adipose tissue may obscure bony landmarks and makes it difficult to accurately determine the insertion site and depth to ligamentum flavum.^19^ One cross-sectional study found that ultrasound-measured depth to the ligamentum flavum increased with BMI (from 44 mm in BMI <25–64 mm in BMI ≥30; *p* < 0.0001).^19^ Meanwhile, there is a strong correlation between ultrasound-measured depth and needle depth required to reach CSF (*r* = 0.803, *p* = 0.000).^20^ In addition, ultrasound was able to identify the L4-L5 spinous processes and spinal canal in 76% of patients with difficult-to-palpate bony landmarks, and difficult palpation was more common with increasing central adiposity.^19^ Without ultrasound, clinicians must rely on surface anatomy to estimate insertion site and depth to the ligamentum flavum, which may be inaccurate in those with increased adiposity. In fact, the literature suggests these factors to be a major risk factor for failed LP attempts.^3^

One RCT that included patients with obesity or difficult anatomy found that USLP significantly improved first-attempt success (80% vs 35%) compared with landmark-guided LPs, with a NNT of 2.2.^21^ The ultrasound group also had a significantly shorter median time to successful LP (*p* = 0.002) and a lower relative risk of traumatic LPs (RR = 0.33, 95% CI 0.08–1.46, *p* = 0.235), although this difference did not reach statistical significance.^21^ A similar study in patients with BMI ≥25 kg/m^2^ reported higher overall success rates with USLP (92.7% vs 68.3%, *p* = 0.011), as well as shorter procedure times (17.02 vs 37.63 minutes, *p* < 0.05) and fewer needle redirections (*p* = 0.017).^15^ Ultrasound guidance remained independently associated with higher LP success after adjusting for BMI (as a continuous variable) and history of lumbar back disease (defined as lumbar scoliosis, L3-S1 spinal stenosis with or without surgery, lumbar arthritis, or lumbar spondylosis) using multivariable logistic regression. USLP also resulted in fewer reports of significant pain (14.6% vs 58.5%) and radicular pain (9.8% vs 29.3%).^15^

These findings highlight the value of ultrasound in identifying anatomy, selecting appropriate needle length, and reducing failed attempts and patient discomfort. Reflecting this, the Society of Hospital Medicine now recommends USLP for patients with increased adiposity at the LP site, prior lumbar surgery, variable interspinous processes, spinal curvature, degenerative bony change, reduced spinal flexibility, or other anatomic variations, reinforcing its growing relevance in clinical practice.^10^

## Limitations of Negative Studies of USLP

Although many studies support the benefits of USLP, some have found no significant benefit compared with landmark-based LPs. However, many of these studies have limitations that may account for these null findings. A common limitation was a small sample size. Two studies ended early due to a low enrollment of 35 patients, resulting in an underpowered analysis.^14,22^ Others tended to have a low representation of patients with obesity, the group that is believed to benefit the most from USLP.^23,24^ For example, one study noted that it did not sufficiently power its subgroup of patients with obesity.^23^ Another study with a population of mean BMI 22.9 ± 4.9 kg/m^2^ reported a nearly 100% success rate in both groups, which is not reflective of actual clinical practice, limiting generalizability.^24^

Minimal or unstandardized USLP training was also a common feature. In 2 studies, novice operators with variable LP and ultrasound experience received only a single 30-minute USLP training session or brief bedside instruction.^22,23^ This raises the possibility that limited experience with ultrasound-guided technique, rather than the technique itself, may have contributed to the lack of observed benefits. Although baseline proficiency in landmark-based LP may influence procedural performance, effective use of USLP also requires familiarity with ultrasound image acquisition and interpretation, which was not consistently developed in these studies. Finally, a recent meta-analysis of RCTs of neuraxial procedures including USLP found no significant improvement with their primary outcome, a first pass success without needle redirection. Although different ultrasound-assisted neuraxial procedures share the goal of using ultrasound to identify spinal anatomy to guide needle insertion, these results were derived from heterogeneous procedures and may be less applicable to USLP specifically. In addition, this study was limited by heterogeneity in operator skill, encompassing both procedural (LP experience) and ultrasound proficiency, as well as terminology across studies. Furthermore, they still found that ultrasound reduced the number of skin punctures, needle redirections, and traumatic LPs.^25^

## Reliance on Fluoroscopic LPs

Despite the growing evidence supporting USLPs, this shift has not been widely adopted within the field of neurology. Instead, an increasing number of LPs are being referred to radiology, often for fluoroscopic guidance. Between 2004 and 2017, the proportion of LPs performed by radiologists rose from 37.1% to 54%, while those performed by neurologists declined from 23.5% to 10%, reflecting a notable shift away from bedside LPs in neurology.^26^

Although radiology referrals may improve success in select complex cases and may appropriately reflect patient complexity, institutional workflow, or safety considerations, routine dependence on radiology for diagnostic LPs can delay clinical decision-making and treatment initiation and increase patient length of stay. A referral to radiology for fluoroscopically guided LP represents an escalation from standard bedside LP, often requiring patient transport and scheduling of a procedure room, which may compete with other image-guided procedures that cannot be done at bedside. This has been cited as a source of delay in care, with 1 study of image-guided procedures (including thoracentesis, paracentesis, and LP) demonstrating that referral to radiology was associated with an approximately 41% increase in time to procedure compared with bedside procedures, corresponding to an average delay of nearly 3 additional hours.^27^ These delays in LP may contribute to worse outcomes, particularly in time-sensitive conditions.^5^ The same study also reported an association between radiology referral and increased health care costs, with a nearly 38% cost increase compared with bedside procedures, although this analysis was not specific to LPs alone.^27^ In addition, fluoroscopy-guided LPs expose patients to ionizing radiation^28^ and often require patients to lie prone, which may be intolerable for those with increased intracranial pressure, pain, or altered mental status.^29^ Referred LPs can also burden radiology residents, reducing their availability for interpreting imaging studies.^30^

The growing reliance on radiology for fluoroscopic LPs is likely multifactorial. Patient specific anatomic considerations may contribute to procedural difficulty,^3,31^ while declining procedural competence among clinicians due to lack of training may limit the ability to manage these cases at the bedside.^32^ Notably, 1 study in Hospital Medicine found that training clinicians on a paramedian USLP technique resulted in a decrease in radiology consults for image-guided LP following unsuccessful bedside attempts (25.0% vs 12.4%, CI −23.7 to −2.8).^18^ Incorporating USLP training in neurology settings may empower neurologists to independently perform challenging LPs that would otherwise require a referral to radiology, improving the efficiency of neurologic care.

## Future Directions

Previous work and recent expert commentary suggest a need for improved approaches to training neurology residents in LP.^3,4,15^ USLP may have potential to address this gap but is limited by lack of research within neurology and limited exposure during neurology training. The Accreditation Council for Graduate Medical Education (ACGME) defines competency benchmarks for procedural skills such as LP but does not mandate standardized curricula for meeting those benchmarks. Instead, ACGME Neurology Milestones recognize image-guided LP (such as with ultrasound) as a level 5 skill, which goes beyond expectations for residency graduation (level 4).^33^ Other international postgraduate medical training organizations such as the Joint Royal Colleges of Physicians Training Board (United Kingdom)^34^ and Royal College of Physicians and Surgeons of Canada^35^ similarly list LP as a competency goal, but do not explicitly reference USLP. As such, awareness and adoption of the technique are limited, and current clinician expertise has been cited as a barrier to training future neurologists in USLP.^36^ A national needs assessment across neurology departments may help clarify current LP and USLP training practices, procedural competency and confidence, exposure to and interest in USLP, and institutional protocols for handling difficult LPs.

Additional RCTs of USLP in neurology settings are required to determine whether the findings from emergency medicine and anesthesiology are applicable to neurology practice. In addition to measuring success rates, insertion attempts, time to insertion, traumatic LPs, and pain scores, which have been the focus of most USLP studies, it would also be valuable to measure the effect of USLP implementation on system-level factors, such as cost, radiology referral rates, time from LP order to successful LP including radiology referral delays, and learner confidence. Although most USLP studies have used a midline approach, alternative techniques such as a paramedian approach have been described^18^ and warrant further comparative study.

Point-of-care ultrasound use is expanding across multiple areas of neurology, including cerebrovascular imaging, procedural guidance, and neuromuscular ultrasound.^37^ As such, there is growing interest in the diagnostic and procedural applications of ultrasound in neurologic practice.^37,38^ In neurocritical care, lack of confidence and training are common barriers to ultrasound use despite strong interest in expanding ultrasound training in the field.^38^ In addition, broader system-level barriers to implementation such as access to ultrasound equipment, availability of trained faculty, and time constraints within clinical workflow have been described in other ultrasound applications and merit further evaluation in neurology.^39-42^

We identified that in some RCTs, limited training without assessment of competency in the USLP group is a potential limitation for interpreting the results. It may strengthen future studies to implement competency-based medical education (CBME) in USLP training. Unlike traditional models that assume competence based on curriculum completion, CBME requires learners to demonstrate predefined competencies before advancing.^43,44^ Using a CBME training approach has shown to greatly improve procedural competency in LP (95.7% mean checklist score) compared with the traditional apprenticeship model (65.4% mean checklist score, *p* < 0.001).^45^ Indeed, they found no relationship between clinical experience and performance on the skills assessment.^45^ One evidence-based model of procedural medical education that incorporates CBME is Sawyer's 6-step framework: “Learn, See, Practice, Prove, Do, and Maintain.”^46^ This framework has been well-received by clinical educators, with 93% of those introduced to the model reporting that they found it helpful for teaching.^47^ As an example, a USLP curriculum following the Sawyer framework may include videos/readings, a live demonstration, practice using an ultrasound-compatible LP phantom (Figure), a required observation and checklist approval from a supervisor, and refresher workshops.

## Limitations

As a narrative review, this study did not use a systematic search strategy and, therefore, may be subject to selection bias in the studies included. Much of the available evidence on USLP was derived from emergency medicine settings, which may limit the generalizability of these findings to the field of neurology. The findings of this review are based on studies in adult populations and may not be generalizable to pediatric patients. In addition, definitions of a “difficult” LP varied across studies and may include differences in BMI thresholds, increased adiposity at the LP site, and criteria used to define spinal anatomical variations such as prior surgeries, reduced spinal flexibility, spinal curvature, or degenerative changes. Finally, publication bias may favor studies demonstrating positive procedural outcomes.

## Conclusion

Ultrasound-assisted LP shows clinical benefits, particularly in patients with obesity or spinal anatomical variations. However, despite evidence from other specialties, its role in neurology remains incompletely defined. Extrapolating from the limited neurologic literature regarding USLP, we hypothesize this technique is both understudied and underutilized within neurologic practice. Expanding investigation within neurology-specific settings will be important to better characterize its effect on procedural success, efficiency, and patient outcomes. Clarifying this role may help guide integration of USLP into clinical neurology and improve procedural skills in bedside LP.

## Glossary

ACGME: accreditation council for graduate medical education
CBME: competency-based medical education
LP: lumbar puncture
NNT: number needed to treat
RCT: randomized controlled trial
USLP: ultrasound-assisted lumbar puncture
